# Summary of the National Advisory Committee on Sexually Transmitted and Blood-Borne Infections (NAC-STBBI) Statement: Recommendations on Screening for Syphilis in Non-Pregnant Adults and Adolescents

**DOI:** 10.14745/ccdr.v50i78a01

**Published:** 2024-07-24

**Authors:** Housne Begum, Stephan Gadient, Jared Bullard, Jennifer Gratrix, Troy Grennan, Todd Hatchette, Annie Fleurant-Ceelen

**Affiliations:** 1 National Advisory Committee on Sexually Transmitted and Blood-Borne Infections Secretariat, Public Health Agency of Canada, Canada; 2 National Advisory Committee on Sexually Transmitted and Blood-Borne Infections Syphilis Working Group, Canada

**Keywords:** screening for syphilis, recommendations, non-pregnant adults and adolescents

## Abstract

**Background:**

Sustained and significant increases in Canadian rates of infectious syphilis prompted the National Advisory Committee on Sexually Transmitted and Blood-Borne Infections (NAC-STBBI) to update the existing screening recommendation for non-pregnant adults and adolescents.

**Methods:**

These guidelines were developed following the 2014 World Health Organization Handbook. The research question was: “What is the clinical utility of syphilis screening using risk-based versus population-wide approaches for adolescents and adults?” The evidence was assessed using the Grading of Recommendations Assessment, Development and Evaluation (GRADE) approach.

**Results:**

The environmental scan included 11 guidelines on syphilis screening published between 2014 and January 2023. Two systematic reviews were identified and included. In the updated literature search from November 6, 2019, to January 17, 2023, there were no published systematic reviews on the effectiveness of risk-based screening or the comparison of risk-based and interval screening; however, one recent randomized control trial in Canada was published. Evidence for outcomes, patient values and preferences, resources, acceptability, equity, cost and cost effectiveness and feasibility were reviewed.

**Conclusion:**

This statement provides two screening recommendations for adults and adolescents. Recommendation 1: NAC-STBBI recommends syphilis screening in all sexually active persons with a new or multiple partners and/or upon request of the individual. They also recommend screening every three to six months in individuals with multiple partners. Recommendation 2: NAC-STBBI recommends that targeted “opt-out” screening programs should be considered as frequently as every three months when serving population groups and/or communities experiencing a high prevalence of syphilis (and other STBBI). Both are strong recommendations with moderate certainty of evidence.

## Introduction

Syphilis is a sexually transmitted infection (STI) caused by the organism *Treponema pallidum* subspecies pallidum and can have significant morbidity if left untreated. In 2020, the World Health Organization (WHO) estimated that 7.1 million new syphilis infections occurred globally (1). Infectious (primary, secondary and early latent stages) and congenital syphilis are on the rise in Canada. Other high-income countries, such as the United States (US), Australia and the United Kingdom have reported similar trends (2–4).

Syphilis is the third most reported STI in Canada, but over the past decade (2013–2022) rates have increased by 393.1%, compared to 33.1% and 181.7% increases in rates for chlamydia and gonorrhea, respectively. The national rate of infectious syphilis increased from 5.1 cases per 100,000 population in 2011 to 24.6 per 100,000 population in 2019 and 36.1 cases per 100,000 population in 2022 (5,6). While rates have historically been higher in males than in females, reported rates of infectious syphilis have been increasing faster among females. Between 2010 and 2019, the rate in females increased by 1,446.8% compared to a 287.9% increase in the rate in males (5). As of January 2020, all provincial/territorial jurisdictions have declared increased rates of infection. The majority of cases continue to be among gay, bisexual and other men who have sex with men (gbMSM), but an increase has been reported in the heterosexual population with the most significant increase being in women of childbearing age, leading to increases in rates of congenital syphilis (6,7).

Sustained and significant increases in Canadian rates of syphilis prompted the National Advisory Committee on Sexually Transmitted and Blood-Borne Infections (NAC-STBBI) to prioritize the review and update of the Public Health Agency of Canada’s (PHAC) existing screening recommendation. Screening is defined as the testing of asymptomatic individuals.

## Methods

Syphilis screening recommendations were developed following the methods outlined in the 2014 edition (8) of WHO handbook for guideline development. A working group (WG) for guideline development comprising four members of NAC-STBBI was established and supported by PHAC secretariat. A methodologist and a team of systematic reviewers from the PHAC STBBI Guidance for Health Professionals Section (PHAC team) independently conducted a systemic review (SR) update of major studies on syphilis screening and scanned previously published syphilis screening guidelines using Google, the websites of international organizations, provincial/territorial organizations and a SR in 2022 by Canada's Drug Agency (CDA-AMC), formerly Canadian Agency for Drugs and Technologies in Health (CADTH) (9). The PHAC SR team examined studies published between January 2010 and January 2023 on syphilis screening, patient values and preferences, equity, feasibility, acceptability, economic analyses and health technology assessments. The evidence was assessed using the Grading of Recommendations Assessment, Development and Evaluation (GRADE) approach.

The WG identified the key questions that formed the basis for the SR and the recommendations as follows:

• Population: adolescents and adults

• Intervention: risk-based screening for syphilis (screening based on clinician assessment and opinion for syphilis with serologic testing using traditional or reverse sequence algorithms)

• Comparator: population-wide screening, at any time interval (e.g., three months, six months, 12 months) for syphilis with serologic testing using traditional or reverse sequence algorithms known as Interval screening

• Outcomes: clinical utility (e.g., incidence of infectious/non-infectious syphilis, neurosyphilis or congenital syphilis), proportion of participants who receive unnecessary or inadequate treatment (e.g., due to false positive/negative test results), participant acceptability and safety (e.g., adverse events, psychosocial harms)

• Study designs: health technology assessments, systematic reviews, randomized controlled trials (RCTs) and non-randomized studies

An environmental scan on existing syphilis screening recommendations of different organizations was conducted. The PHAC SR team also searched for SRs, then primary studies when no SRs were available. Evidence for outcomes, patient values and preferences, resources, acceptability, equity and feasibility were reviewed from published and unpublished literature. Comprehensive searches for previously conducted SR, RCTs and non-randomized studies were performed in September 2019 and updated in January 2023. Two members of the PHAC SR team screened studies, extracted and analyzed the data and assessed the quality/certainty of the evidence using the GRADE approach (10). A total of 11 guidelines on syphilis screening published between 2014 and January 2023 were reviewed (11–21). The most common screening intervals were every three to six months. The Appraisal of Guidelines for Research & Evaluation (AGREE) II instrument (22) was used to evaluate the methodological quality of the identified guidelines. From a literature search with the Health Canada Librarian in 2019, two systematic reviews (23,24) were identified and included.

The updated literature search from November 6, 2019, to January 17, 2023, with the librarian resulted in 220 records. After removal of duplicates, there were a total of 176 articles. The WG members shared four additional articles and one more was found in an article reference list. After title and abstract screening, 31 records were included for full text screening and a final total of nine records were included. There were no published SRs on the effectiveness of risk-based screening or the comparison of risk-based screening with interval screening; however, one RCT was published (25). There were two more updated SR findings included from CDA-AMC (9) and the US Preventive Services Task Force (USPSTF) (26). Of the 1,032 search records found by CDA-AMC, only one overview of reviews by Fernane and Fowler (27) met the pre-specified inclusion criteria focusing on screening adult patients (16 years of age and older) at low risk for syphilis (27). The updated search by the USPSTF included one study by Chow *et al.* (28) on screening effectiveness. In addition, 10 studies were included from the librarian’s search, hand search and suggested citations from the WG members on “risk-based screening vs. interval screening”, “comparison of annual, three months and six-month screening intervals”, “syphilis screening as part of HIV [human immunodeficiency virus] viral load testing” and “opt-in vs. opt-out approach.”

## Results

The evidence review included three SRs (23,24,27) and 11 studies on syphilis screening: one randomized (25) and 10 non-randomized studies, including three cohort studies (29–31), seven retrospective chart reviews and cross-sectional studies (see **Appendix** for Evidence Profiles, **Table A1**) (28,32–37). The certainty of the evidence for the screening of syphilis was moderate. An environmental scan of 11 guidelines on syphilis screening published between 2014 and January 2023 was completed (11–21). All organizations recommend risk-based screening. Four organizations recommend screening for those at increased risk of infection at varying intervals, from annual screening to up to four times a year depending on risk behaviours. The most common intervals were every three to six months.

From PHAC search results, one RCT (25) reported that in risk-based screening versus interval screening, the average annual number of syphilis tests per individual increased from 0.53 to 2.02 tests and the time-adjusted rate ratio was 2.03 (1.85–2.22) (25). With intervention, the annualized proportion of newly identified early syphilis increased from 0.009 to 0.032 and the odds of annual screening increased nearly four-fold while the mean number of tests per year increased two-fold (25). Comparison of annual, three and six-month screening intervals during routine serology taken as part of HIV monitoring resulted in a marked increase in the proportion of HIV-positive men who have sex with men (MSM) diagnosed with asymptomatic syphilis (28,29,32,33,37). Additional studies using modelling projected similar results (38,39). These studies showed that increasing the frequency of syphilis screening to every three months was the most effective strategy for reducing infectious syphilis cases.

Targeted screening was more effective than universal screening as part of HIV viral load testing when using the opt-out strategy (30). Over 50.8% of incident syphilis cases were asymptomatic and were only identified through routine screening (30). One observational study compared risk-based screening, opt-in and opt-out approaches for HIV-positive gbMSM (31). The authors found that the opt-in (opt-in means offering syphilis testing to HIV-positive MSM and conducting the test in those that agree, which may be related to their perceived risk) and opt-out (opt-out refers to syphilis testing done automatically on all HIV-positive MSM unless a patient declines to have the test) approaches led to increased uptake of syphilis testing. A risk-based testing approach (risk-based involves assessing risk and then offering a syphilis test accordingly) resulted in lower testing frequencies and potentially missed opportunities (31). Reekie *et al.* (34) also examined the uptake of opt-out versus opt-in screenings in a remand facility in Alberta, Canada, between March 1, 2018, and February 28, 2020, among individuals younger than 35 years. They found that the opt-out approach screened more admissions among those younger than 25 years, even though the total opt-out uptake was low (n=902/2,906; 31.2%). Opt-in screenings achieved significantly high positivity rates for syphilis. Opt-out screening resulted in higher STI positivity rates compared to other STIs (chlamydia, gonorrhea) (29.5%), however, lower than rates from opt-in screening (35.8%). Both found similar HIV-positivity rates (34).

Another study in the US (35) found a large number of missing cases while targeting screening to only those deemed “high-risk” by behaviour or symptoms. Venegas *et al.* (30) also found opt-out screening using technology and risk factors identified 27 of the 59 patients with reactive syphilis tests considered newly diagnosed syphilis infection (no history of syphilis infection reported in the system) and requiring follow-up treatment.

A qualitative study reported on patient values and preferences, feasibility and equity for syphilis screening in males accessing HIV care (40). Most males were in favour of routinely testing for syphilis as part of conventional HIV care. The routine method was thought to have a destigmatizing effect on syphilis testing. From the patient’s point of view, HIV care clinics are easy locations to be tested for syphilis. Reekie *et al.* reported (34) the feasibility of opt-out screening in a short-term correctional facility for individuals younger than 35 years in Alberta, Canada. They reported that opt-out screening at admission is feasible and can improve STI testing in high-risk individuals experiencing incarceration in Canada (34,40).

Four cost effectiveness modelling studies examining either risk-based screening or interval screening were included (41–44). The modelling studies were based in Canada, the US, Germany and Australia. The studies did not directly compare the cost effectiveness of risk-based screening to interval screening for syphilis. Studies also focused primarily on high-risk population groups, such as gbMSM, people living with HIV and sex workers. Generally, targeted screening at three or six-month intervals was considered more cost-effective compared to universal annual screening in these populations (41–44).

## Recommendations

Following the review of available evidence, NAC-STBBI recommends the following two recommendations for healthcare professionals. Recommendations developed by NAC-STBBI are made at the population level. It is important to note that they may not apply to specific individuals within those groups, particularly as it relates to groups and communities who may have higher rates of syphilis when compared to the general public. It is always essential to consider each case on an individual basis in the context of the risk behaviours and epidemiological factors outlined in the recommendation. The full statement contains a more detailed explanation of the recommendations, dissemination, implementation, monitoring and evaluation.

### Syphilis screening for sexually active adults and adolescents

NAC-STBBI recommends syphilis screening in all sexually active persons with a new or multiple partners and/or upon request of the individual. NAC-STBBI recommends screening every three to six months in individuals with multiple partners. **(*Strong recommendation, moderate certainty of evidence*)**

### Syphilis screening for high prevalence groups/communities

NAC-STBBI recommends that targeted opt-out screening programs should be considered as frequently as every three months when serving population groups and/or communities experiencing high prevalence of syphilis (and other STBBI), such as gbMSM, people living with HIV, people who are or have been incarcerated, people who use substances and/or access addiction services and/or some Indigenous communities. **(*Strong recommendation, moderate certainty of evidence*)**

Screening programs should consider aligning screening with other health services (“opportunistic screening”) for individuals living with HIV and other individuals at increased risk accessing care services. Opportunistic screening is defined as offering screening when an individual is accessing non-emergency health services and has not undergone recent STBBI testing.

Screening programs should consider local epidemiology when determining which groups/communities to target and for a specific individual, travel history and patient risk factors need to be considered.

## Discussion

When determining who to screen for syphilis and other STBBIs, providers should consider the individual risk factors for the person seeking care. Nurses and physicians therefore must discuss these factors with the individual to determine their sexual health history and identify the appropriate screening tests. Unfortunately, many individuals may not feel comfortable discussing their sexual health due to stigma and/or prior poor experience with the healthcare system. Additionally, individuals will often underestimate their own personal risk when it comes to STBBI. To address these challenges, healthcare providers are encouraged to consider implementing strategies such as an opt-out approach to screening, thereby removing the need for an in-depth discussion on the person’s sexual history. These programs have experienced greater success compared to opt-in programs in certain settings. Applying opt-out programs can further normalize STBBI screening and help reduce the discomfort and, more importantly, stigma related to sexual health.

Healthcare providers should also consider offering screening when patients are accessing other non-emergency healthcare services to increase instances of STBBI screening. Opportunistic screening for STBBI is a mechanism healthcare providers should consider implementing for individuals with limited or infrequent access to care. Regardless of whether the individual is there for STBBI-related care, healthcare providers should take the opportunity to determine when they last underwent STBBI screening and offer it as appropriate. Screening can occur as frequently as every three months for individuals who engage in behaviours that increase their risk level (e.g., multiple partners) or are part of a high prevalence population (e.g., people who use substances). Importantly, normalizing and standardizing the offering of STBBI screening can help mitigate and reduce the perception of stigma.

Healthcare providers must also be aware of the increasing rates of congenital syphilis across Canada. There were 117 cases of confirmed congenital syphilis in 2022, compared to only eight cases in 2017, representing an increase of more than 1,300%. Additionally, cases of infectious syphilis among females increased by 720% over that span (6,42). It is essential that healthcare providers be mindful of these trends when providing care to females of childbearing age (approximately ages 15–45 years) to ensure the proper STBBI screening is offered. Care providers are reminded that universal STBBI screening is recommended in all pregnant people.

It should be noted that much of the evidence used to develop these recommendations were focused on gbMSM populations and individuals living with HIV. Considering that gbMSM populations continue to have higher rates of STBBI infections compared with other communities and that individuals living with HIV are at increased risk of acquiring other STBBI, the recommendations may overestimate the frequency of screening needed in the public. Additionally, the rapidly changing epidemiology has resulted in significant change to the incidence and prevalence of syphilis, which can result in certain studies becoming quickly outdated when the population being assessed no longer reflects the population being impacted by the bacteria. Ongoing review and monitoring of the most up-to-date surveillance data is integral to ensure individuals/populations with high infection prevalence are identified quickly.

Prioritizing STBBI research on the general public should be considered given studies focused on the general population are lacking and can result in a gap in the evidence. Extrapolating evidence from these groups to apply to the general population is not always feasible given significant differences in population groups and their respective risk factors.

## Conclusion

Recent increases in rates of infectious syphilis and congenital syphilis can be addressed and mitigated through proper screening. It is important for healthcare providers to be aware of the growing public health burden of syphilis so that cases can be identified, treated and the onward transmission of the infection interrupted. Overall, NAC-STBBI recommends that syphilis screening should be offered to all sexually active persons with a new or multiple partners and/or upon request of the individual. NAC-STBBI recommends that screening should be offered every three to six months in individuals with multiple partners. They also agreed that targeted opt-out screening programs should be considered as frequently as every three months for health services serving population groups and/or communities experiencing a high prevalence of syphilis (and other STBBI). The certainty of the evidence for the screening of syphilis is moderate.

## Appendix

**Table A1:** Evidence profiles

Question 1: Should [risk-based approaches] vs. [population wide/interval screening approaches] be used for [syphilis screening among sexually active adolescents and adults]?	
Outcome	Evidence
**Risk-based screening vs. interval screening**	
Syphilis screening Number of serological tests performed (1 RCT) ((25))	Average annual number of syphilis tests per individual increased from 0.53 to 2.02 tests Time-adjusted rate ratio: 2.03 (1.85–2.22)
		Untreated early syphilis cases diagnosed (1 RCT) ((25))	With intervention, the annualised proportion of newly identified early syphilis increased from 0.009 to 0.032
Annual screening (1 RCT) ((25))	The odds of annual screening increased nearly 4-fold
Certainty of evidence	⨁⨁⨁◯^a^ MODERATE Imprecision
**Comparison of annual, 3-month and 6-month screening intervals**	
Number/proportion of serological tests performed (5 observational studies) ((28,29,32,33,37))	The inclusion of routine syphilis serology taken as part of HIV monitoring resulted in a marked increase in the proportion of HIV-positive MSM diagnosed with asymptomatic syphilis
Certainty of evidence	⨁⨁⨁◯ MODERATE^b,c^ Risk of bias
Projected number of reported incident syphilis cases from studies using modelling ((38,39))	Increasing the frequency of syphilis screening to every three months was the most effective strategy for reducing infectious syphilis cases Focused screening was more effective than universal screening Enhanced screening of MSM with prior syphilis may efficiently reduce transmission, especially when identification of high-risk men via self-reported partner numbers or high-frequency screening is difficult to achieve
**Opt-in vs. opt-out approach**	
Diagnosed higher new syphilis cases (4 observational studies) ((31,34–36))	Opt-out screening: Diagnosed higher new syphilis cases (case-finding rate). Opt-out: 7.3% (150/2,053 tests); opt-in 7.1% (150/1,995 tests) Number of syphilis tests per man increased from 1.3 in 2006 to 2.2 in 2007 (*p*<0.01) In 2010, the proportion of men having ≥3 syphilis tests in a year was highest in the clinics with the opt-out strategy (48%; range: 35%–59%), compared to the opt-in (39%, *p*=0.12) and risk-based (8.4%; range: 5.4%–12%, *p*<0.01)
Certainty of evidence	⨁⨁⨁◯ MODERATE^b,c^ Risk of bias
**Syphilis screening as part of HIV viral load testing**	
Syphilis tests on the same day as HIV viral loads (1 observational study) ((30))	In 2010, same day tests was highest in clinics with the opt-out strategy (87%; range: 84%–91%), compared with opt-in (74%, *p*=0.121) and risk-based (22%; range: 20%–24%, *p*<0.01)
Certainty of evidence	⨁⨁◯◯ LOW^a,b,c^ Risk of bias, imprecision
Number of syphilis tests (1 observational study) ((30))	Over 50.8% of incident syphilis cases were asymptomatic and were only identified through routine screening
Certainty of evidence	⨁⨁◯◯ LOW^a,b,c^ Risk of bias, imprecision

Abbreviations: MSM, men who have sex with men; RCT, randomized control trial

^a^ Total number does not meet the optimum sample size

^b^ One arm of the study was considered and the authors did not mention any information related to the use of an appropriate analysis method that adjusted for all the critically important confounding domains

^c^ It was a retrospective study and the authors did not mention any information related to the use of an appropriate analysis method that adjusted for all the critically important confounding domains
